# Mucosal Immunization of Cynomolgus Macaques with the VSVΔG/*Z*EBOVGP Vaccine Stimulates Strong Ebola GP-Specific Immune Responses

**DOI:** 10.1371/journal.pone.0005547

**Published:** 2009-05-14

**Authors:** Xiangguo Qiu, Lisa Fernando, Judie B. Alimonti, P. Leno Melito, Friedericke Feldmann, Daryl Dick, Ute Ströher, Heinz Feldmann, Steven M. Jones

**Affiliations:** 1 Special Pathogens Program, National Microbiology Laboratory, Public Health Agency of Canada, Winnipeg, Manitoba, Canada; 2 Department of Immunology, University of Manitoba, Winnipeg, Manitoba, Canada; 3 Department of Medical Microbiology, University of Manitoba, Winnipeg, Manitoba, Canada; New York University School of Medicine, United States of America

## Abstract

**Background:**

*Zaire ebolavirus* (ZEBOV) produces a lethal viral hemorrhagic fever in humans and non-human primates.

**Methodology/Principal Findings:**

We demonstrate that the VSVΔG/ZEBOVGP vaccine given 28 days pre-challenge either intranasally (IN), orally (OR), or intramuscularly (IM) protects non-human primates against a lethal systemic challenge of ZEBOV, and induces cellular and humoral immune responses. We demonstrated that ZEBOVGP-specific T-cell and humoral responses induced in the IN and OR groups, following an immunization and challenge, produced the most IFN-γ and IL-2 secreting cells, and long term memory responses.

**Conclusions/Significance:**

We have shown conclusively that mucosal immunization can protect from systemic ZEBOV challenge and that mucosal delivery, particularly IN immunization, seems to be more potent than IM injection in the immune parameters we have tested. Mucosal immunization would be a huge benefit in any emergency mass vaccination campaign during a natural outbreak, or following intentional release, or for mucosal immunization of great apes in the wild.

## Introduction

Ebola virus (EBOV) and Marburg virus (MARV) are members of the *Filoviridae* family causing a lethal viral haemorrhagic fever (HF). *Zaire ebolavirus* (ZEBOV), is the most virulent species in the Ebola genus with human case fatality rates of over 80%, [1]–[5]. Sporadic outbreaks of ZEBOV have occurred in Central Africa since 1976 causing more than 1,800 human cases, as well as epidemics among chimpanzees and great apes [6]–[9]. Populations of wild apes in Central Africa have been decimated and there is a significant risk that these populations could become extinct because of ongoing outbreaks [7]. Importantly in the bordering region between Gabon and the Republic of Congo, human outbreaks have been shown to correspond to outbreaks in great apes, and so preventing disease in these animals could potentially prevent many human cases.

Experimental infections of non-human primates (NHPs) with ZEBOV by intramuscular injection or mucosal exposure results in a disease with symptomology similar to humans [10]–[12]. Transmission of ZEBOV is generally through exposure to infected bodily fluids via either breaks and abrasions of the skin, or through mucosal surfaces. It is currently unknown which plays a more prominent role in the spread of disease. However, infection can occur via aerosol administration [13]. In addition, there is evidence that ZEBOV can be transmitted between experimentally inoculated monkeys and naïve controls probably by virus-laden droplets secreted or excreted leading to mucosal infection [14]. For this reason there is concern of EBOV being incorporated into a biological weapon. In addition, research was conducted into the use of weaponized MARV with the most likely means of dispersal through aerosol generation [15]. The severity and high fatality of the disease, in addition to the possibility of EBOV or MARV to be used as a bioterrorist weapon has necessitated the research and development of vaccines for both EBOV and MARV. Currently there is no preventative vaccine or post-exposure treatment option approved for human or animal use.

Early attempts at creating a vaccine included γ-irradiated MARV [16] or ZEBOV [10]; or formalin-inactivated ZEBOV [17]. These vaccines were able to provide protection in guinea pigs, but in the NHP model the vaccines failed to protect. Recent attempts focusing on DNA and viral based vaccines, or virus like particles (VLP) have been more promising. These newer vaccines are more effective in several animal models however, to date only five EBOV vaccine platforms have demonstrated over 80% efficacy in the more stringent NHP model [18]–[26]. Previously, we described the generation and use of live attenuated recombinant Vesicular Stomatitis Virus (VSV) with the VSV glycoprotein replaced by the glycoprotein of ZEBOV (VSVΔG/ZEBOVGP) or MARV (VSVΔG/MARVGP) [27]. Furthermore, we demonstrated that these live attenuated recombinant VSV vaccines completely protect NHPs against lethal challenge with the corresponding filoviruses by intramuscular injection (IM) [25], [26], [28]. In this study we are investigating for the first time the protection provided by intranasal (IN) or oral (OR) immunization with the VSV-based vaccines against the standard systemic IM challenge with ZEBOV in NHPs. We investigated the humoral and cellular immune responses following vaccination as well as the long term memory B and T cell immune responses post-challenge.

## Results

### Clinical Observations

**Figure 1:**
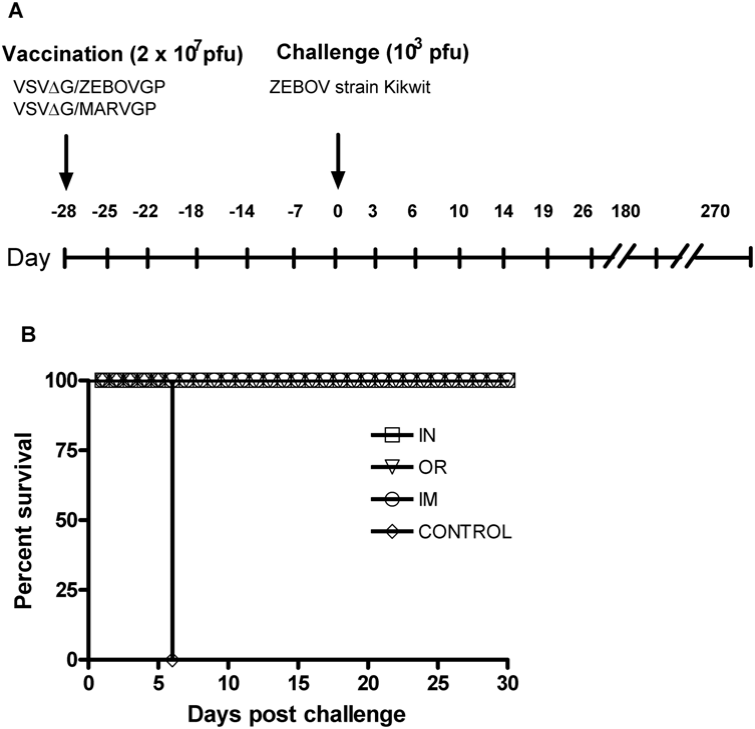
Survival of vaccinated cynomolgus macaques. A) *Flow chart of experimental design.* Arrows indicate vaccination and challenge date. Tick marks indicate sampling days. B) *Kaplan-Meier survival curve for cynomolgus macaques immunized by different routes and challenged with Zaire ebolavirus*. Cynomolgus macaques were immunized orally (OR; n = 4), intranasally (IN; n = 4) or intramuscularly (IM; n = 2) with 2×10^7^ PFU of VSVΔG/ZEBOVGP, or injected intramuscularly with VSVΔG/MARVGP (control; n = 2). All animals were challenged 28 days later with 1000 PFU ZEBOV. The animals were scored daily for fever, macular rashes, lethargy, and unresponsiveness.

We used 12 cynomolgus macaques, of which 10 were immunized with VSVΔG/ZEBOVGP either orally (OR; n = 4), intranasally (IN; n = 4) or intramuscularly (IM; n = 2). The remaining 2 control animals were vaccinated intramuscularly with VSVΔG/MARVGP. VSVΔG/MARVGP does not provide any heterologous protection against ZEBOV, therefore these NHPs succumb to ZEBOV infection. All animals were challenged with 1000 PFU of ZEBOV 28 days later, and monitored daily for clinical symptoms. An IM challenge was chosen as we have previously demonstrated that it produces a rapid and lethal ZEBOV HF [25]. An aerosol mucosal delivery mechanism was not employed as the disease course progressed more slowly, and we wished to test the vaccine against the most severe disease [13]. In addition, we have already demonstrated that the VSVΔG/ZEBOVGP vaccine fully protects against an aerosolized lethal dose of ZEBOV [13]. In this current study, all of the VSVΔG/ZEBOVGP immunized animals were protected from the high dose challenge and showed no evidence of clinical illness after vaccination or ZEBOV challenge. However, the control animals demonstrated typical symptoms associated with ZEBOV HF, such as fever, macular rashes, lethargy, unresponsiveness and were euthanized on day 6 (Figure 1). We performed haematology analysis at each examination date, and other than increases in the platelet-crit in the OR and IN groups post-challenge, we saw no significant changes in any NHPs post-immunization or in the VSVΔG/ZEBOVGP immunized NHPs post-challenge (Table 1). Due to the high variability across the 2.5 months of sampling it was difficult to determine any significance within or between groups. However, there was a trend towards decreased lymphocytes and platelets that does correspond to previous reports.

**Table 1:** Blood Hematology Results for Cynomolgus Monkeys after vaccination and challenge with Ebola Virus.

Parameter	VSVG/ZEBOVGP						Controls	
	OR		IN		IM		VSVG/MARVGP	
	Post Vac	Post Chall	Post Vac	Post Chall	Post Vac	Post Chall	Post Vac	Post Chall
WBC	NC	NC	NC	NC	NC	NC	NC	NC
% Lymphocytes	NC	NC	NC	NC	NC	NC	↑ day 10*	↓ day 3*
% Monocytes	↑ day 3 (2/4)*	NC	NC	NC	NC	NC	↑ day 3 & 6*	NC
%Neutrophils	NC	NC	NC	NC	NC	NC	NC	NC
%Eosinophils	NC	NC	NC	NC	NC	NC	NC	NC
%Basophils	NC	NC	NC	NC	NC	NC	NC	NC
HCT	NC	NC	NC	NC	NC	NC	NC	NC
RBC	NC	NC	NC	NC	NC	NC	NC	NC
HGB	NC	NC	NC	NC	NC	NC	NC	NC
Reticulocytes	NC	NC	NC	NC	NC	NC	NC	NC
% Reticulocytes	NC	NC	NC	NC	NC	NC	NC	NC
MCV	NC	NC	NC	NC	NC	NC	NC	NC
% RDW	NC	NC	NC	NC	NC	NC	NC	NC
MCHC	NC	NC	NC	NC	NC	NC	NC	NC
MCH	NC	NC	NC	NC	NC	NC	NC	NC
Platelets	NC	NC	NC	NC	NC	NC	NC	NC
MPV	NC	NC	NC	NC	NC	NC	NC	NC
PCT	NC	↑ day 6–26 (3/4)*	NC	↑ day 10,14,19 (3/4)*	NC	NC	NC	NC
% PDW	NC		NC	NC	NC	NC	NC	NC

Pre-vaccination test results for each animal (n = 12) were used to establish an average for each parameter. Post-vaccination and -challenge values were compared to the pre-vaccination average. Significant changes in comparison to this average are indicated (*). When values for each animal were compared to its' own pre-vaccination values, no changes (NC) were observed in any of the parameters.

HCT = hematocrit, RBC = red blood cells, HGB = hemaglobin, MCV = mean corpuscular volume, % RDW = red cell distribution width, MCHC = mean corpuscular hemoglobin concentration, MCH = mean corpuscular hemoglobin, MPV = mean platelet volume, PCT = platelet-crit, % PDW = platelet distribution width. Post Vac = post vaccination, Post Chall = post challenge, OR = oral, IN = intranasal, IM = intramuscular.

### Evaluation of the antibody response

**Figure 2:**
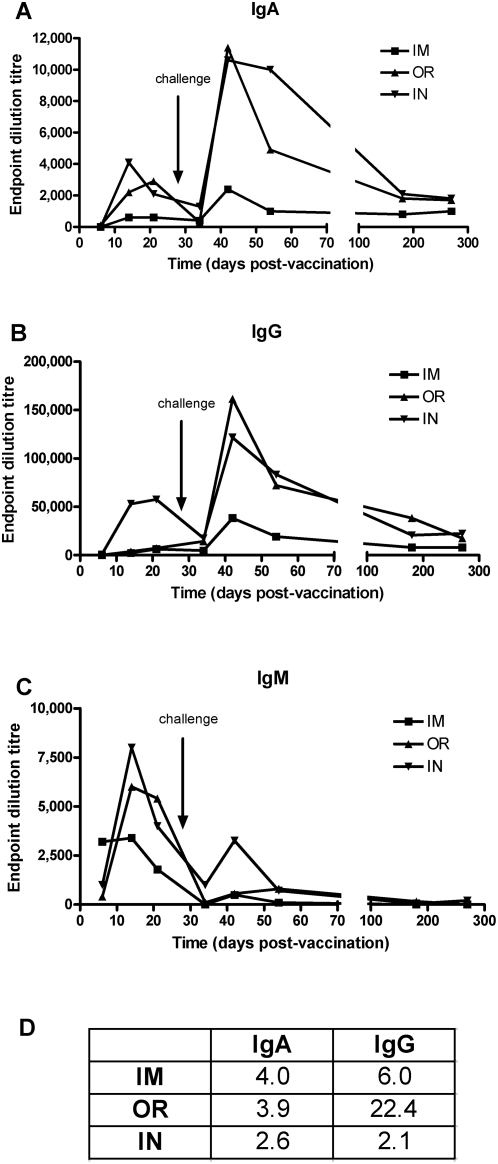
Strong humoral IgM, IgA, IgG responses are induced in the IM, OR, and IN immunization routes. A ZEBOV VLP ELISA was utilized to determine the ZEBOV-specific IgA (a), IgG (b), IgM (c) titres in post-vaccination and post-challenge sera from cynomolgus macaques immunized either orally (OR; n = 4), intranasally (IN; n = 4) or intramuscularly (IM; n = 2). Titres are presented as endpoint dilutions of the average value per group. Table (d) illustrates fold increases in peak titres post-challenge compared to post-vaccination for IgA and IgG. IgM is not shown as there were no increases post-challenge.

To characterize the humoral immune response, a virus like particle (VLP) based ELISA was used to determine the levels of ZEBOVGP-specific antibodies. The VSVΔG/MARVGP immunized animals never developed a detectable antibody response to ZEBOVGP. In contrast, potent antibody responses were detected in all VSVΔG/ZEBOVGP immunized animals independent of immunization route (Figure 2). Between days 14 and 21 post-vaccination, all VSVΔG/ZEBOVGP immunized NHPs developed high levels of IgA (IM = 1∶600; OR = 1∶2,900; IN = 1∶4,100), IgM (IM = 1∶3,400; OR = 1∶6,000; IN = 1∶8,000), and IgG (IM = 1∶6,400; OR = 1∶7,200; IN = 1∶57,600) against ZEBOVGP. After challenge the IgM titres did not exceed the post-vaccination levels, however, IgG and IgA antibody titres were significantly increased peaking 14 days post-challenge then slowly decreasing before maintaining a relatively high antibody titre up to 9 months. Overall in the post-vaccination response, the IgM titres ranked IN≅OR>IM, with an IN titre 2.4 times higher than IM, while IgA and IgG responses ranked IN>OR>IM (IN 6.8 fold >IM) and IN>OR≅IM (IN 9.0 fold >IM), respectively.

**Figure 3:**
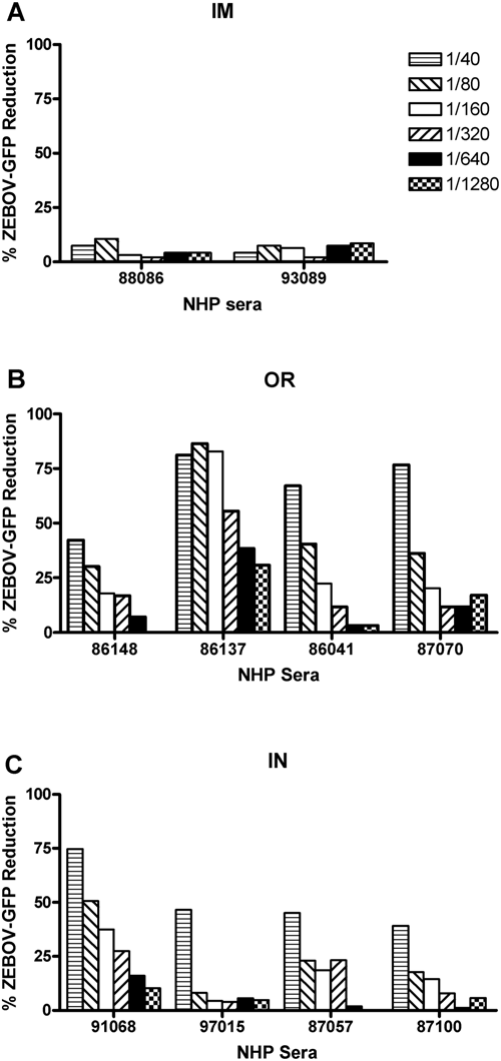
Neutralizing antibodies generated by the various immunization routes. ZEBOV GP-specific neutralizing antibodies in the sera from NHPs vaccinated either (A) intramuscularly (IM), (B) orally (OR), or (C) intranasally (IN), were investigated for their ability to inhibit infection by ZEBOV-GFP. NHP sera from days 0 and 21 post-vaccination were incubated for 1 hour with ZEBOV-GFP before being added to a monolayer of Vero cells. The positive control was ZEBOV-GFP in DMEM without sera. The level of GFP fluorescence of ZEBOV-GFP infected cells was determined by flow cytometry. The percent reduction of infection by ZEBOV-GFP was calculated as follows: % ZEBOV-GFP reduction = (1−(Test samples/Positive control))×100). The day 21 results for each NHP are displayed and have been normalized by subtracting each NHP's day 0 results from the day 21 results. The numbers in the legend represent the dilution of the NHP sera that was added to ZEBOV-GFP.

In order to determine the level of neutralizing antibodies (NAb) present in the sera 21 days post-vaccination, a ZEBOV-GFP neutralization assay was performed (Figure 3). The ZEBOV-GFP virus contains the GFP between the NP and VP35 gene [29]. Although the ZEBOV-GFP virus is slightly attenuated in *in vivo* NHP studies, the growth kinetics in the Vero E6 cell line is virtually indistinguishable from the wild type ZEBOV, suggesting similar levels of infectivity [29], [30] The IM immunization produced low but detectable levels of NAb (Figure 3a). In comparison, 3/4 NHPs in the OR group (Figure 3b) demonstrated a 50% reduction in ZEBOV-GFP positive cells at a titre of 1∶40 (mean = 66.7%, range:42.3–81.1%). One of the OR immunized NHPs had a 55.5% reduction at the 1∶320 dilution. Similarly, the IN route (Figure 3c) resulted in a reduction of ZEBOV GFP positive cells at the 1∶40 dilution (mean = 51.4%, range:39.1–74.8%). Similar results were obtained using the standard plaque neutralization assay (data not shown). Overall, while the OR and IN routes produced ZEBOVGP-specific Nabs, the NAb titres are relatively low, with the OR route producing the highest titres post-vaccination

### Evaluation of the cellular immune response

**Figure 4:**
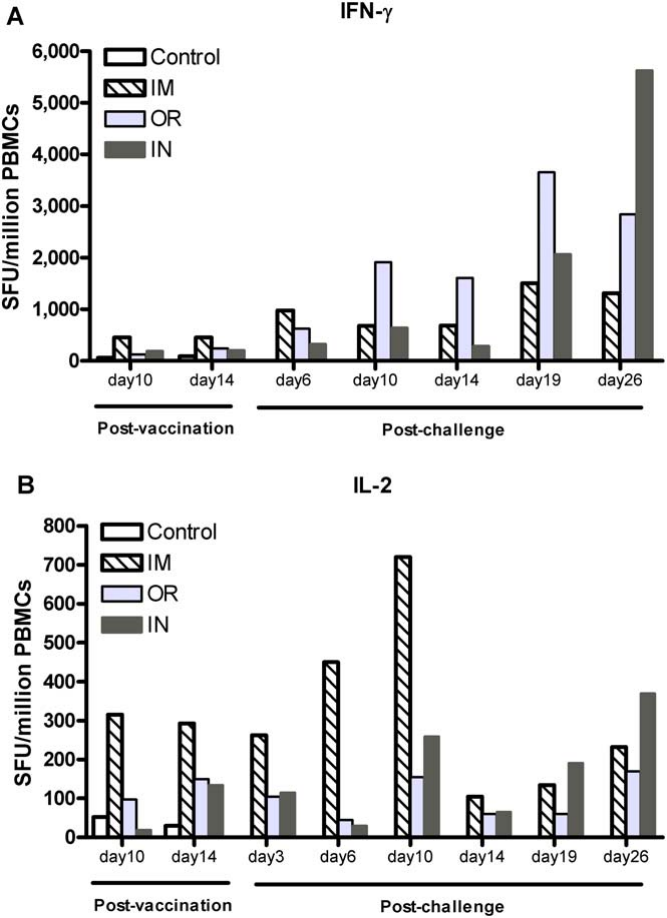
PBMCs from immunized animals produce IFN-γ or IL-2 in response to ZEBOVGP peptides. ZEBOVGP-specific IFN-γ or IL-2 secreting cells from PBMCs were detected by either an (A) IFN-γ or (B) IL-2 ELISPOT assay. PBMCs were obtained from cynomolgus macaques immunized with VSVΔG/ZEBOVGP either orally (OR; n = 4), intranasally (IN; n = 4), or intramuscularly (IM; n = 2). The control NHPs (n = 2) were immunized IM with the heterologous VSVΔG/MARVGP. PBMCs were incubated with 1.5 µg/ml of peptides spanning the entire ZEBOV glycoprotein. Bars represent the average number of ZEBOVGP-specific IFN-γ or IL-2 secreting cells detected in each immunized group.

To evaluate the ZEBOVGP-specific effector cellular immune responses, IL-2 and IFN-γ ELISPOT assays were conducted to determine the number of IL-2 and IFN-γ secreting lymphocytes (Figure 4). IFN-γ is produced by activated T lymphocytes and NK cells. Prior to challenge on days 10 to 14 post-vaccination there was a detectable ZEBOVGP-specific IFN-γ response in all immunized animals (Figure 4a). The IM route was the most potent, inducing approximately 2 fold more IFN-γ secreting cells than OR (p<0.001) or IN (p = 0.043) routes. However, post-challenge a strong secondary IFN-γ response was induced in all VSVΔG/ZEBOVGP immunized animals with the IM route producing the most IFN-γ cells at day 6 but by day 10 was overtaken by a stronger response in the OR group. The IFN-γ in the IN group rose steadily, peaking at day 26 post-challenge with 4.3 and 2 fold more ZEBOVGP specific IFN-γ secreting cells than the IM (p = 0.003) and OR (p = 0.075) group, respectively. All three routes produced strong ZEBOVGP-specific IFN-γ responses, with the IM route dominant early after challenge but surpassed by the OR and IN routes at later time points.

Another T-cell cytokine, IL-2, plays a key role during infection by inducing proliferation/activation of CD4^+^ and CD8^+^ T cells, potentiates the cytotoxicity of CD8^+^ T lymphocytes and Natural Killer (NK) cells, and stimulates B lymphocyte function. Post-vaccination, the IM group had more ZEBOVGP-specific IL-2 secreting cells than either of the mucosally immunized groups (Figure 4b). This difference was significant for the IN route on day 10 (p<0.001) and 14 (p = 0.022). Post-challenge the IM route continued to dominate early after challenge peaking on day 10. This difference shows a trend when compared to the IN group (p = 0.067) and is significant when compared to the OR group (p<0.001). Additionally, the IN group had more IL-2 producing cells than the OR group (p = 0.090) on day 10 post-challenge. By day 26 post-challenge all three routes continued to produce a ZEBOVGP-specific IL-2 response, however the IN group response was strongest with a ranking of IN>IM≅OR (IN vs. IM p = 0.051; IM vs. OR p = 0.838). At day 26 post-challenge the IN group had the most potent IFN-γ and IL-2 responses, as well as the highest IgA and IgG antibody titre, indicating this immunization route, followed by a ZEBOV challenge, results in the development of potent and sustained effector responses.

### Absolute white blood cell counts following ZEBOV challenge

**Figure 5:**
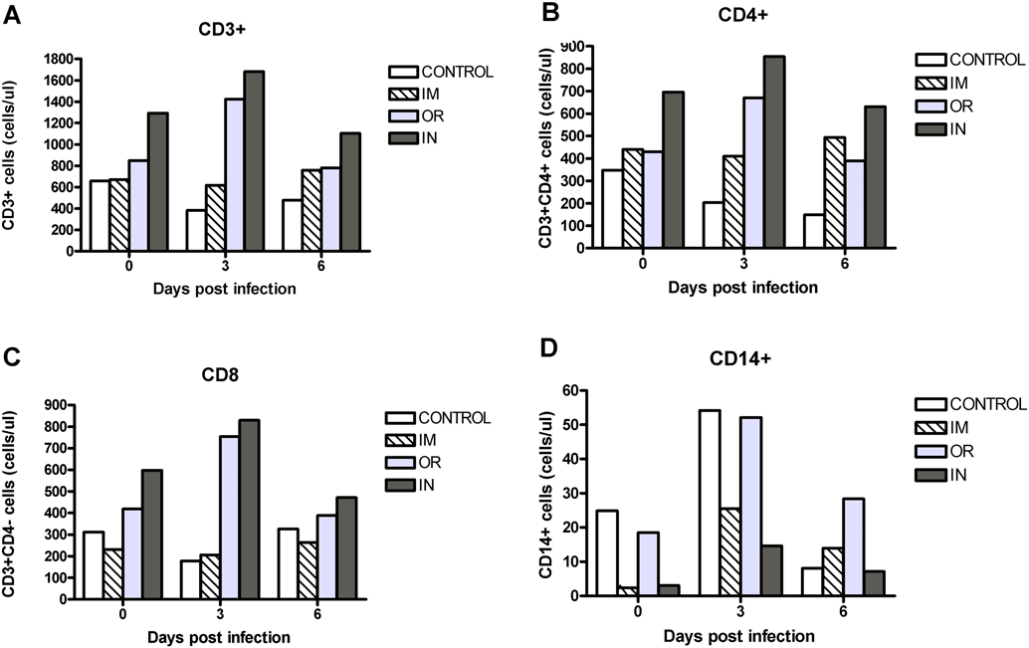
Absolute white blood cell numbers do not decrease in immunized animals after Zaire ebolavirus challenge. Whole blood from cynomolgus macaques immunized either orally (OR; n = 4), intranasally (IN; n = 4) or intramuscularly (IM; n = 2) was stained for lympocytes (CD3^+^, CD4^+^, CD8^+^), monocytes (CD14^+^). Control animals (n = 2) received non protective VSVΔG/MARVGP. The absolute numbers in blood was determined for the each of the monkeys by flow cytometry on days 0, 3 and 6 post-challenge with ZEBOV. The average for each inoculation route is represented.

In immunized animals a secondary immune response should occur upon ZEBOV challenge resulting in a rapid amplification of lymphocytes. However, previous reports on ZEBOV infections in unvaccinated animals demonstrate a decrease in lymphocytes possibly due to apoptosis [30]–[32]. In order to compare the changes in cell numbers between the immunization routes, and also confirm that lymphocytes were not lost in vaccinated animals following ZEBOV challenge, absolute lymphocyte numbers for CD3^+^, CD4^+^, and CD8^+^ (CD3^+^4^−^) T cell populations were determined by flow cytometry (Figure 5). As expected, in the VSVΔG/MARVGP (control) vaccinated animals who are not protected from ZEBOV, the lymphocyte numbers decreased 28–57% (Figure 5a: CD3^+^:day 3 ↓0.58×, p = 0.001; day 6 ↓0.72×, p = 0.015. Figure 5b:CD4^+^:day 3 ↓0.58×, p = 0.003; day 6 ↓0.43×, p = 0.002. Figure 5c:CD8^+^:day 3 ↓0.57×, p = 0.003, day 6 ↑1.04×, p = 0.642) on days 3–6 compared to day 0 post-challenge data. In contrast, there was no decrease in the lymphocyte populations for any of the VSVΔG/ZEBOVGP vaccinated NHPs (Figures 5a–c). Comparison within VSVΔG/ZEBOVGP inoculation routes demonstrated no significant changes from day 0 in either the IM or IN groups, although numbers of both CD4^+^ (Figure 5b) and CD 8^+^ (Figure 5c) T cells did increase on day 3 post-challenge in the IN group (CD4^+^: day 3 IN ↑1.22×, p = 0.433; CD8^+^: day 3 IN ↑1.39×, p = 0.053). The most substantial increases in total CD3^+^, CD4^+^, and CD8^+^ numbers were on day 3 (Figure 5a:CD3^+^: day 3 ↑1.68×, p = 0.032; Figure 5b:CD4^+^: day 3 ↑1.56×, p = 0.093; Figure 5c:CD8^+^: day 3 ↑1.80×, p = 0.025) in the OR group, that then returned to normal levels by day 6. VSVΔG/ZEBOVGP vaccination prevented the loss of lymphocytes seen in non-protected NHPs. In fact the OR and IN routes showed increased numbers of lymphocytes on day 3 post-challenge probably indicating a proliferation of ZEBOVGP-specific T-cells.

As antigen presenting cells, the macrophages have a major role in the initiation of the immune response to foreign antigens. However, they are also the primary cells infected by ZEBOV early during infection [33], [34]. Therefore, the number of CD14^+^ macrophage/monocytes was examined post-challenge (Figure 5d). The number of CD14^+^ cells increased 2.2× (p = 0.190) on day 3 in the control group, but the numbers were greater in the VSVΔG/ZEBOVGP vaccinated groups with the IM route showing the most significant increases (IM ↑10.9×, p = 0.001; OR ↑2.8×, p = 0.062; IN ↑4.9×, p = 0.009).

### ZEBOVGP Specific Memory Responses

**Figure 6:**
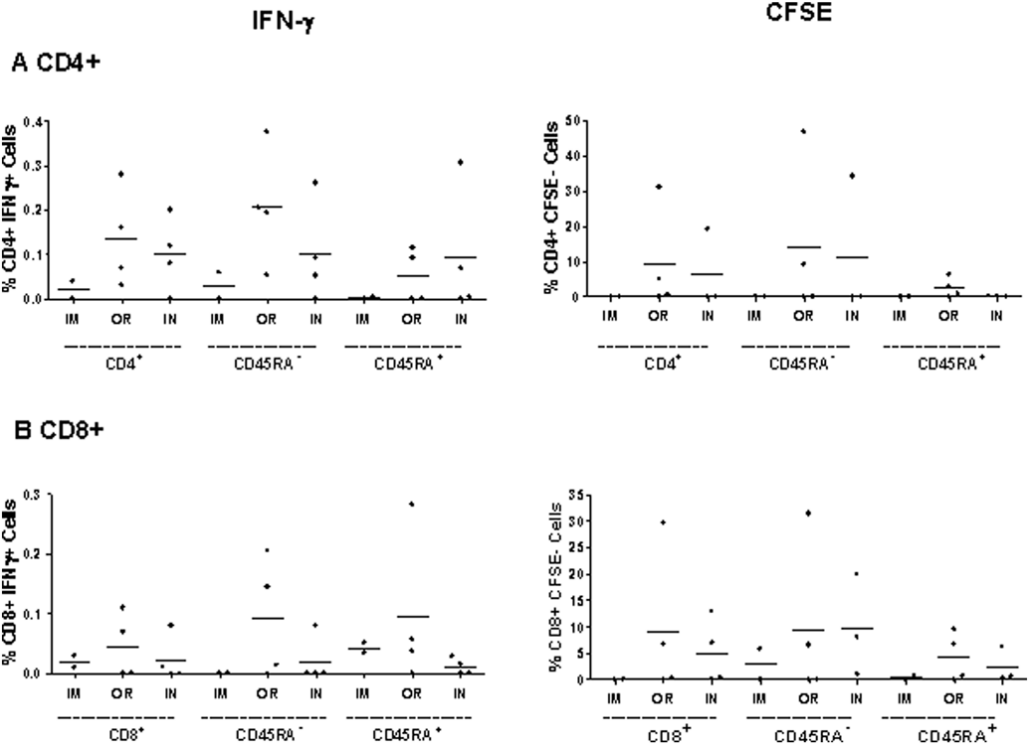
ZEBOVGP-specific CD4^+^ and CD8^+^ functional memory responses in PBMCs. Flow cytometry was utilized to evaluate the long term CD4+ (A) and CD8+ (B) ZEBOVGP-specific functional memory responses of freshly isolated PBMCs incubated with media or ZEBOVGP peptides at 6 months post-vaccination. The average for each inoculation route (IM = intramuscular n = 2, OR = orally n = 4, IN = intranasally n = 4) is represented. The CD45RA^−^ population denotes the memory lymphocytes, while CD45RA+ represents the naïve and effector lymphocytes. The IFN-γ response was evaluated after 3 days by intracellular cytokine staining. The background from the media sample was subtracted from the peptide stimulated sample, and the results are shown for each monkey with the average for each group being represented with a bar. The ability of the PBMCs to proliferate in response to the GP peptide was determined by staining the PBMCs with CFSE and then looking for loss of CFSE 6 days later as a measure of proliferation. The percent of CFSE^−^ cells for each memory population is shown for each monkey after subtracting the media sample, and the average for each group is represented by a bar.

In order to determine the long term immune response after challenge, ZEBOVGP-specific CD4^+^ (Figure 6a) and CD8^+^ (Figure 6b) memory T lymphocytes were examined for their ability to proliferate (CFSE^−^) or produce IFN-γ in response to ZEBOVGP peptides at 6 months post-vaccination. The T lymphocyte marker CD45RA was used to determine the memory population (CD45RA−), and the naïve plus effector populations (CD45RA+). In the IM immunized group, there was a very low level of proliferation in the CD8+ memory population accompanied by low levels of IFN-γ production in the naïve/effector CD8+ populations, as well as the CD4+ memory population. In comparison, the OR and IN immunized groups saw higher levels of proliferation and IFN-γ production in their CD4^+^ and CD8^+^ memory cells. The memory cells in both the OR and IN groups proliferated equally well however the OR group had a higher percentage of memory cells producing IFN-γ than the IN group. The CD45RA^+^ naïve/effector populations also demonstrated low levels of proliferation as well as produced IFN-γ in response to the ZEBOVGP peptides in the OR and IN groups. This is presumed to be primarily due to the effector population and not the naïve cells. Overall, the ZEBOVGP-specific memory responses that occur as a result of vaccination followed by a ZEBOV challenge persist for at least 6 months indicating that long term immunity is possible in filovirus infections. The fact that the memory populations in OR and IN inoculation routes demonstrate the greatest potential for proliferation and IFN-γ production post-challenge suggests that different routes of immunization prime different memory cell populations.

## Discussion

In this study we have completed the only long term immune response study, and the most extensive investigation of the T-cell memory responses yet conducted with ZEBOV infected NHPs. This data forms a foundation for future critical studies on the immune correlates of protection, the potential for rapid mass vaccination programs in humans, and the immunization of endangered great ape populations in Africa.

We demonstrated previously that a single IM dose of VSVΔG/ZEBOVGP protects cynomolgus macaques from a high dose challenge with ZEBOV [25]. In this study, we explored potential routes of immunization to compare the protection conferred as well as the immune response each route would elicit. All immunization routes were equivalently efficacious as no NHPs died or displayed symptoms of HF. In all cases both ZEBOVGP-specific humoral and cell mediated immune responses (CMIR) were generated, although some routes generated more potent humoral responses. All animals demonstrated a high IgM response post-vaccination, which was not exceeded following challenge. This response is normal since IgM is produced upon initial exposure to a new antigen during the primary but not the secondary immune response. In general the post-vaccination IgM and IgA titres were the highest in the IN group followed closely by the OR group, but 2.4 and 6.8 fold lower, respectively, in the IM route. This suggests that IN immunization was effective in eliciting not only a primary immune response but also IgA antibodies important for mucosal immunity. Importantly, no adverse effects were seen in any of the immunized animals.

The most crucial immunoglobulin is IgG since it provides the majority of the systemic antibody, which is important for a haematological infection of ZEBOV. Once again the IN route was most effective with post-vaccination titres 8–9 fold higher than with OR and IM routes. With ZEBOV challenge, the secondary immune response IgG titres increased 43, 16 and 2 fold for OR, IM and IN, respectively. Although IN titres only increased 2 fold, the final titres were similar to the OR route as the IN pre-challenge titres were high initially, and the OR immunized animals had a very strong secondary IgG immune response after challenge. Very significantly the IM route consistently resulted in lower IgG titres before and after challenge when compared to the mucosally immunized animals. However, the IM animals all survived and this may indicate a minimum threshold of antibody required for protection, with all three routes achieving this minimum. In contrast to the total IgG titres, the NAb titres were systemically very low (titres = 1∶40 to 1∶320) and seem unlikely to be important as a protective mechanism, which is consistent with previous reports. In murine studies and other NHP studies, anti-ZEBOV NAb were low or absent yet the animals were resistant to a ZEBOV challenge [35]–[37].

The CMIR is a vital protective mechanism against viral pathogens and in this study was induced by all immunization routes. ZEBOV infections result in the loss of peripheral blood lymphocytes, presumably due to apoptosis [30]–[32], [38]. In this study, absolute lymphocyte counts rather than percentages were used to determine whether the VSVΔG/ZEBOVGP vaccine was able to prevent their decline. This is very important since changes in percentages can be caused by increases or losses in cell populations other than the one under observation. Absolute counts in contrast measure a true value for the number of circulating cells under investigation and other population changes do not effect the measurement. While the controls demonstrated the typical loss in lymphocytes, after challenge, all vaccination routes displayed a relatively stable or mild increase of CD3^+^, CD4^+^ and CD8^+^ numbers. An increase in lymphocyte numbers is typical of the expansion seen during a secondary immune response, and was most pronounced for the OR administration.

Further evidence to support the induction of CMIR is the production of IFN-γ and IL-2 cytokines measured by ELISPOT. Previous studies have investigated the production of TNFα by T cells rather than IL-2 however, TNFα does not have a specific role in the induction or maintenance of the CMIR and was therefore not included in this study. IFN-γ secretion by T lymphocytes, NKs, and dendritic cells, occurs early in the immune response to a pathogen, and promotes the activation of antigen presenting cells as well as the induction of a Th1 response. IL-2 which is produced by T lymphocytes during an immune response, stimulates the growth, differentiation and survival of antigen-specific cytotoxic cells. All three routes induced the production of both IL-2 and IFN-γ. Post-vaccination and very early post-challenge the IM route induced the most IFN-γ and IL-2 secreting cells. However, cytokine production in the OR and IN routes increased steadily post-challenge and by day 26 post-challenge the OR and IN cytokine secreting cells surpassed the IM group. The production of IFN-γ post-vaccination contrasts a previous study were we had demonstrated that IFN-γ was not produced in peripheral blood mononuclear cells (PBMCs) post-vaccination [25]. The discrepancy could be due to the different assays and tissues used, as intracellular cytokine staining of PBMCs by flow cytometry was performed in the earlier study versus the ELISPOT assay on the peripheral blood cells in this paper. Overall, in this paper, the strong production of IL-2 and IFN-γ by IM injection early on suggests the ability to induce a strong Th1 CMIR.

The induction of long term immunity is a key requirement of any vaccine. In this study we examined the immune response induced by the vaccines up to 28 days, as well as the long term immunity that occurred after vaccination plus challenge. The immune responses directly attributable to the vaccine demonstrate that the OR and IN vaccination produce stronger humoral responses while the IM route provides a stronger CMIR. After challenge the strength, type and duration of the immune responses were also measured. The ZEBOVGP-specific T lymphocyte memory response and antibody titre was examined at 6 and 9 months post-vaccination, respectively. The OR and IN administration produced long lasting CD4^+^ and CD8^+^ memory lymphocytes that were able to proliferate and produce IFN-γ in response to ZEBOVGP peptides. On the other hand the IgA and IgG serum titres were high for all three routes with a ranking of IN>OR>IM. Based on this data it suggests that different routes of immunization prime the memory responses differently. These differences are reflected in the memory responses seen after challenge in regards to the strength and type of response. The IM route is more likely to produce a strong Th1 CMIR while the IN route induced a predominantly Th1 humoral response. The potent CMIR seen in the IM group may be a product of the high IFN-γ and IL-2 production detected post-vaccination, and early on post-challenge. IFN-γ has immunoregulatory activities during an immune response to viral pathogens, and in addition it has antiviral effects that may inhibit early viral replication and thereby slow the infection [39]. Although the memory responses elicited at 6 and 9 months do not represent the effectiveness of the vaccine alone, this measurement indicates the route of immunization has an impact on the strength and type of immune response that is initiated upon a subsequent infection. In addition it demonstrates that long term protection to filoviruses is achievable, and provides us with clues as to the correlates of immune protection.

The immune correlates of protection have not been defined for the protection of NHPs against filoviruses. Previously, we demonstrated an 80% survival in mouse adapted ZEBOV (MA-ZEBOV) infected mice that had received VSVΔG/ZEBOVGP immune sera, indicating that the humoral response is partially responsible for protection [40]. NAb titres in vaccinated animals, or humans that have survived EBOV infections have consistently been absent or low (1∶20 to 1∶180) [41], [42]. Therefore a non-NAb response appears to be the mechanism involved in the humoral response to filovirus infections. In addition, all mice immunized with VSVΔG/ZEBOVGP survived challenge with MA-EBOV even when depleted of CD8^+^ lymphocytes, suggesting that the cytotoxic T lymphocyte response may not be required [40]. As further confirmation, PBMCs from NHPs that had been immunized with an EBOV GP plus NP DNA prime then Adenovirus ZEBOV GP boost were examined for their ability to proliferate to ZEBOV peptides. When the CD4^+^ population had been depleted the ability to proliferate in response to the peptides was reduced to background levels, but when the CD8^+^ population was depleted there was no drop in proliferation [22].

In our system, total Ab response, and the pre-challenge IgG titres achieved from all routes of immunization with the VSVΔG/ZEBOVGP vaccine were high (1∶6,400 to 1∶57,600). Although there is no evidence for the importance of IgA, it is vital for mucosal immunity especially in the event of an aerosol delivery of ZEBOV [43]. All routes were able to produce high IgA titres although the IN and OR delivery was most effective. In addition, the OR route was able to induce a detectable CD4^+^ CMIR which also appears to be vital to survival. Development of a vaccine for OR or IN delivery would be desirable because they can be self administered significantly reducing the requirement for trained personnel especially in areas where the virus is endemic; they stimulate mucosal immune responses, which play an essential role in protecting the lungs from aerosol exposure; and they are more accepted by patients as there is no needle.

We have shown conclusively that mucosal delivery of the vaccine can protect from IM systemic challenge and very surprisingly mucosal delivery particularly IN immunization seems to be more potent than IM injection in many immune parameters. Mucosal immunization would be huge benefit in any emergency mass vaccination campaign for natural outbreaks or following intentional release. Furthermore, mucosal immunization may be of great significance if immunization of great apes is attempted in the wild.

## Materials and Methods

### Ethics Statement

Animal studies were performed in biosafety level 4 biocontainment at Public Health Agency of Canada and approved by the Canadian Science Centre for Human and Animal Health Animal Care Committee following the guidelines of the Canadian Council on Animal Care. Animals were acclimatized for 14 days prior to infection. Animals were fed and monitored twice daily (pre- and post-infection) and fed commercial monkey chow, treats and fruit. Husbandry enrichment consisted of commercial toys and visual stimulation.

### Vaccine vectors, viruses, and peptides

The recombinant VSVΔG/ZEBOVGP and VSVΔG/MARVGP vaccine expressing the glycoproteins of ZEBOV (strain Mayinga) or *Lake victoria marburgvirus* (MARV) (strain Musoke) were generated using VSV (Indiana serotype) as described previously [27], [44]. The ZEBOV (strain Kikwit) challenge virus was passaged in Vero E6 cells prior to challenge, as described previously [25], [45]. A ZEBOVGP1,2 peptide pool consisting of 15mers with 11 amino acid overlaps (Sigma-Genosys) spanning the entire 676 amino acids of the ZEBOV, strain Mayinga 1976 GP was used.

### Animals

Twelve 5 to 19 year old healthy cynomologus macaques (*macaca fascicularis*) were from a Health Canada non-human primate colony (Health Canada Animal Resources Division, Tunney's Pasture, Ottawa, Ontario). Animal studies were performed in biosafety level 4 biocontainment at Public Health Agency of Canada and approved by the Canadian Science Centre for Human and Animal Health Animal Care Committee following the guidelines of the Canadian Council on Animal Care. Animals were acclimatized for 14 days prior to infection. Animals were fed and monitored twice daily (pre- and post-infection) and fed commercial monkey chow, treats and fruit. Husbandry enrichment consisted of commercial toys and visual stimulation.

### Vaccination and Challenge Experiment

Twelve filovirus naïve cynomolgus monkeys randomized into four groups received 2 ml of 1×10^7^ PFU/ml of vaccine in Dulbecco's modified Eagle's medium (DMEM). Animals in the three experimental groups were vaccinated with either: 1) 2 ml orally (OR) (n = 4); 2) 1 ml dripped into each nostril, intranasally (IN) (n = 4); or 3) 1 ml each into two sites intramuscularly (IM) (n = 2). The two controls were injected intramuscularly with 2 ml of 1×10^7^ PFU/ml of VSVΔG/MARVGP. All animals were challenged intramuscularly 28 days later with 1,000 PFU of ZEBOV (strain kikwit).

Routine examination was conducted on 0, 2, 4, 6, 10, 14 and 21 days post-vaccination, then 0, 3, 6, 10, 14, 19, 26 days, 6 and 9 months after the ZEBOV challenge. For the examinations animals were anaesthetized by intramuscular injection with 10 mg/kg of ketaset (Ayerst). Examinations included haematological analysis, monitoring temperature (rectal), respiration rate, lymph nodes, weight, hydration, discharges and mucous membranes. Also, swabs (throat, oral, nasal, rectal, vaginal) and blood samples were collected (4 ml from femoral vein, 1 ml in EDTA vacutainer tube; 3 ml in serum separator vacutainer tube). Cynomolgus monkey PBMCs were isolated using BD CPT sodium citrate Vacutainers (Becton Dickinson) as per manufacturer's protocol.

### ELISA

Generation of ZEBOV VLPs were described previously [46]. High binding polystyrene microtitre plates (Thermo) were coated with 60 µl 5 µg/ml ZEBOV VLPs in phosphate buffered saline (PBS) for I hour, 37°C. Plates were washed 4× with PBS, 0.1% Tween-20, then blocked with 200 µl PBS-2% skim milk overnight, 4°C, before sera diluted in blocking solution (60 µl) was added, in triplicate, for 1 hour, 37°C. After washing 4×, 60 µl of detection antibodies (Peroxidase-conjugated goat-anti-monkey, IgG, IgM, or IgA) diluted 1∶2000 in blocking solution were added for 1 hour, 37°C. After washing 4×, 100 µl of substrate (ABTS+H_2_O_2_) was added for 30 minutes at room temperature, before reading on a Versamax microplate reader at 405 nm. A sample was positive when the absorbance was higher than the mean plus 4 standard deviations of the pre-vaccination negative control from each monkey.

### ZEBOV-GFP Flow Cytometric Neutralization Assay

Serum samples from days 0 and 21 post-vaccination were assessed, in duplicate, for their ability to neutralize an infection with ZEBOV-GFP in VeroE6 cells. Serially diluted serum samples were incubated with an equal volume of ZEBOV-GFP [29] in DMEM, at 37°C, 5% CO_2_ for 1 hr before 150 µl was added per well of a confluent 12 well plate of VeroE6 cells (MOI = 0.0005). After 2 hours at 37°C, 5% CO_2_, 1 ml of DMEM, 2% fetal bovine serym (FBS), 100 U/ml penicillin, 100 µg/ml streptomycin was added per well and incubated for 5 days. Cells were harvested by removing the culture supernatant, washing with 1 ml PBS, 0.04% EDTA, then adding 800 µl of PBS 0.04% EDTA for 5 minutes at 37°C before adding 8 ml PBS, 4% paraformaldehyde (PFA) overnight. The cells were acquired (10,000 events) and analyzed with CellQuest Pro v3.3 on a Becton Dickinson FACSCalibur flow cytometer. The percent reduction of green fluorescent protein (GFP) fluorescence was calculated as follows: Percent GFP reduction = (1−(Test sample/Positive control))×100). The positive control was ZEBOV-GFP and the negative control was DMEM. The day 21 samples were normalized by subtracting each NHPs day 0 value, therefore the limits of detection for this assay are at or below the zero v alue of the y-axis.

### ELISPOT Assay

Human IFN-γ and IL-2 BD ELISPOT assays (BD Bioscience) were performed as per manufacturer's instructions. Briefly, 2×10^5^ PBMCs/well were incubated with 1.5 µg/ml of each ZEBOVGP1,2 peptide. Spots were quantified by an automated ELISPOT reader (CTL, Cleveland, OH). The ELISPOT response was considered positive when the number of specific spots/million PBMCs was over 5 times the number detected in the dimethylsulfoxide (DMSO) control wells.

### Immunophenotyping

Whole blood (100 µl) was stained with a 100 µl mastermix of antibodies (CD4 PerCP Cy5.5, CD14 PeCy7, CD3 APC Cy7). CD8^+^ T cells were identified as the CD3^+^4^−^ population. After 30 minutes, 4°C, red blood cells (RBCs) were lysed with 1.5 ml of 1× FACS lysing solution (Becton Dickinson), vortexed, incubated at room temperature for 10 minutes before centrifugation at 300 g, 10 minutes. The pellet was washed with 1 ml of wash buffer (Dulbecco's PBS, Invitrogen). Cells were pelleted as before and resuspended in 900 µl of PBS, 4% PFA. Samples were acquired (10,000 events) and analyzed on a BD LSRII flow cytometer using FACS Diva software v5.0.2 (Becton Dickinson). Prior to running, 100 µl of Flowcount fluorospheres (Beckman Coulter) were added for determination of absolute counts.

### T lymphocyte ZEBOVGP-specific Memory Response

For the memory response assays, 5×10^5^ PBMCs in 500 µl RPMI supplemented with 10% FBS, 2 mM L-glutamine and 100 U/ml penicillin, 100 µg/ml streptomycin (RPMI-10) were incubated with 1.5 µg/ml ZEBOVGP1,2 peptides, or media, at 37°C, 5% CO_2_ and measured for their ability to produce IFN-γ on day 3, or proliferate on day 6. To observe the IFN-γ response cells were washed twice with PBS, 2% FBS before blocking with 10 ul 1 mg/ml human γ-globulin for 10 minutes at room temperature. Then a 100 µl mastermix of CD45RA PECY5, CD4 APC and CD8 PECy7 was added for 30 minutes, 4°C. Cells were stained for IFN-γ using the intracellular staining protocol of BD's cytofix/cytoperm Plus (with GolgiStop) Kit. Cells were then washed and the pellet resuspended in 1 ml of PBS, 4% PFA. Samples were acquired (100,000 events) and analyzed on the LSRII (Becton Dickinson) using the FACS Diva software v5.0.2.

Cell proliferation on day 6 was monitored by a decrease in carboxyfluorsecein diacetate succinimidyl ester (CFSE) staining. On day 0 of the assay, 2×10^6^ PBMCs in 200 µl PBS were stained with 200 ul of 3 µM CFSE (Invitrogen) for 8 minutes, 37°C. After adding 400 ul of cold FBS for 1 minute, cells were washed 2× with 10 ml of PBS and resuspended at 1×10^6^ cells/ml in RPMI-10. Then 500 µl of cells were combined with either media (negative control) or 1.5 µg/ml ZEBOV GP1,2 peptides. On the sixth day samples were subjected to the same blocking and surface staining as the day 3 samples before washing cells, then resuspending in 1 ml of PBS, 4% PFA. Samples were acquired (30,000 events) and analyzed as above.

### Statistics

The T test was used to determine significant responses for the Elispot and flow cytometry data. A significant result was when p≤0.050.
